# GC-MS Chemical Characterization and Antibacterial Effect of the Essential oil of *Piper mosenii*

**DOI:** 10.3390/molecules27185911

**Published:** 2022-09-12

**Authors:** Ana Valéria de Oliveira Braz, Mariana Carvalho Rodrigues, Philippe Alencar Araújo Maia, Ana Paula Dantas Pereira, Jussara de Lima Silva, Francisco Antonio Vieira dos Santos, Luiz Everson da Silva, Wanderley do Amaral, Maura Lins dos Santos, Henrique Douglas Melo Coutinho, Tomasz Baj, Grażyna Kowalska, Radosław Kowalski, Edinardo Fagner Ferreira Matias

**Affiliations:** 1 Faculdade CECAPE, Juazeiro do Norte 63024-015, Ceará, Brazil; 2 Research Laboratory of Natural Products, Federal University of Paraná—UFPR, Matinhos 80060-000, Paraná, Brazil; 3 Department of Biological Chemistry, Regional University of Cariri—URCA, Crato 63105-010, Ceará, Brazil; 4 Department of Pharmacognosy with the Medicinal Plant Garden, Medical University of Lublin, 1 Chodźki Str., 20-093 Lublin, Poland; 5 Department of Tourism and Recreation, University of Life Sciences in Lublin, 15 Akademicka Str., 20-950 Lublin, Poland; 6 Department of Analysis and Food Quality Assessment, University of Life Sciences in Lublin, 8 Skromna Str., 20-704 Lublin, Poland

**Keywords:** antibacterial agent, bacterial resistance, oral hygiene, mouthwash, essential oils

## Abstract

Commercialized mouthwashes are generally expensive for the most financially vulnerable populations. Thus, several studies evaluate the antimicrobial potential of herbal products, such as essential oils, to reduce the activity of microorganisms in the mouth. The objective of this research was to carry out the chemical characterization and antibacterial activity of the essential oil of *Piper mosenii* (EOPm), providing data that enable the development of a low-cost mouthwash formulation aimed at vulnerable communities. The analysis of the antibacterial potential and modulator of bacterial resistance was verified by the microdilution method to determine the minimum inhibitory concentration-MIC. The chemical components were characterized by gas chromatography coupled to mass spectrometry, where 23 chemical constituents were detected, with α-pinene, being the major compound. The EOPm showed a MIC ≥ 1024 µg/mL for all bacterial strains used in the tests. When the EOPm modulating activity was evaluated together with chlorhexidine, mouthwash and antibiotics against bacterial resistance, the oil showed a significant synergistic effect, reducing the MIC of the products tested in combination, in percentages between 20.6% to 96.3%. Therefore, it is recommended to expand the tests with greater variation of EOPm concentration and the products used in this research, in addition to the evaluation of toxicity and in vivo tests, seeking the development of a possible formulation of mouthwash accessible to the vulnerable population.

## 1. Introduction

Biofilm is one of the etiological factors of dental caries and periodontal diseases and consists of an accumulation of microorganisms immersed in an organic matrix of polysaccharides that adhere to dental enamel surfaces [1]. The oral cavity microbiota is colonized by about 400 to 500 different types of microorganisms, distributed in the oral epithelium, tongue dorsum, dental surface, and gingival sulcus epithelium [2].

Among the most common microorganisms of the oral microbiota, the bacterium *Streptococcus mutans* stands out, considered the main etiological agent of dental caries in humans. Virulence factors, such as the composition of its cell surface and the production of toxins, have been investigated in relation to its cariogenic potential [3]. The bacterium *Staphylococcus aureus*, in turn, is part of the group of Gram-positive cocci and is the most common agent in pyogenic infections and abscesses [2]. *Escherichia coli* belongs to the Gram-negative group and is one of the main bacteria responsible for infectious diseases, as it produces enterotoxins and has effective participation in diarrhea [4].

In order to prevent the dental biofilm from resulting in oral pathologies, it is necessary to have mechanical control, performed mainly with a brush, usually associated with toothpaste and dental floss [5]. However, there are factors such as the patient’s lack of guidance or motivation, which contribute to the fact that he does not perform an effective physical removal, leaving organized niches of biofilm on the surface of the teeth [6]. Therefore, there is a need to use chemical substances, such as mouthwashes, to complement mechanical methods [7].

To have an antimicrobial potential, the chemical agent must have some properties, such as the ability to attenuate the adhesion of bacteria to the dental surface, inhibit the growth of microorganisms, in addition to altering bacterial biochemistry, in order to reduce the formation of cytotoxic products [5]. Among the active compounds most used in mouthwashes are chlorhexidine, cetylpyridine chloride, triclosan and essential oils [6].

Chlorhexidine, from the biguanide group, has a broad action on both gram-negative and Gram-positive bacteria, such as *Streptococcus mutans*, being, therefore, the main and most effective choice for chemical control of dental biofilm, mainly due to its substantivity, and consequently its inhibitory action on glycosidic and proteolytic enzymes [5,6,7,8].

Commercialized mouthwashes are generally expensive for the most financially vulnerable populations. In addition, some chemical compounds when used more frequently, can cause collateral damage, such as changes in the color of teeth and soft tissue [9]. Given these factors, there are several scientific studies that evaluate the potential of herbal products, such as essential oils, to reduce the activity of commensal microorganisms in the oral cavity. In addition to being a more economically viable alternative, essential oils can contribute to the prevention and treatment of biofilm-dependent diseases, in addition to reducing side effects when used correctly, either alone or in combination with other drugs [10].

In this context, plants of the genus *Piper*, belonging to the Piperacea family, stand out for the production of essential oils and have great biological and ethnopharmacological relevance [11,12]. Among the biological potentials described for this genus, the antibacterial, anti-inflammatory, analgesic activities, as well as antitumor activities of some species can be emphasized [13,14]. The *Piper mosenii* C. DC species, popularly known as “pariparoba”, is present in the Southeast regions. and Southern Brazil [15]. The essential oil of this species has chemical constituents that demonstrate antimicrobial activity against some strains of microorganisms, such as *Staphylococcus aureus* and *Candida albicans* [16].

Given this perspective, the research developed aimed to carry out the chemical characterization and antibacterial potential of the essential oil of *Piper mosenii*, providing data that enable the development of a low-cost mouthwash formulation aimed at vulnerable communities.

## 2. Results

After analysis in GC-FID and GC-EM of the essential oil sample of *Piper mosenii* (EOPm), 23 chemical constituents were detected, which corresponded to 87.2% of the total composition of the sample (Table 1). Furthermore, based on this chemical evaluation, α-pinene (No. 1) was the major compound, with 22.8%, followed by myristicin (No. 16), bicyclogermacrene (No. 15), β-caryophyllene (No. 9), with 16.5%, 8.3% and 6.1% respectively; this majority chemical composition is compatible with literature data that describe the constituents of *Piper mosenii* [14].

An evaluation of the antibacterial activity of *Piper mosenii* essential oil showed that EOPm was active against *Staphylococcus aureus*, with a MIC of 512 μg/mL [16]. However, in this research, when analyzing the antibacterial potential of the essential oil of *Piper mosenii* (EOPm), with the determination of the minimum inhibitory concentration (MIC), the EOPm presented a MIC ≥ 1024 µg/mL for all bacterial strains used in the tests. The results of this research indicated that EOPm has no antibacterial potential, as MIC values ≥1024 µg/mL are clinically irrelevant.

When analyzing the modulating activity of bacterial resistance of EOPm combined with antibacterial products for clinical use, namely chlorhexidine, mouthwash, ampicillin, gentamicin and penicillin G, relevant results were obtained, whose values were organized and demonstrated in 1, 2, 3, and 5 figure, so that facilitate understanding.

**Figure 1:**
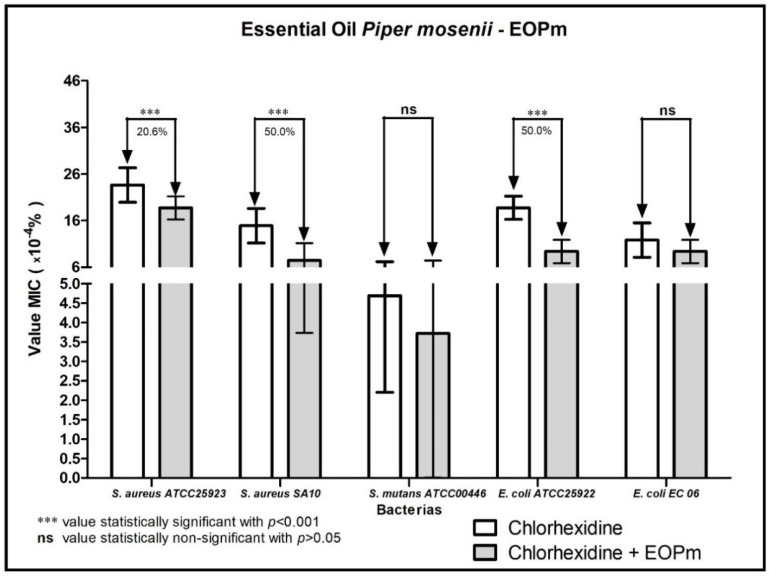
Evaluation of the modulatory activity of EOPm combined with Chlorhexidine against bacterial strains.

The essential oil of *Piper mosenii* when associated with chlorhexidine showed that there was a 50% decrease in MIC against the strains of *S. aureus* SA10 and *E. coli* ATCC25922, while against the strain of *S. aureus* ATCC25923 the reduction of the MIC of chlorhexidine was lower, with a percentage of 20.6%; these results indicate that there was a synergistic effect in the situations evaluated, since EOPm reduced the MIC of chlorhexidine (Figure 1).

**Figure 2:**
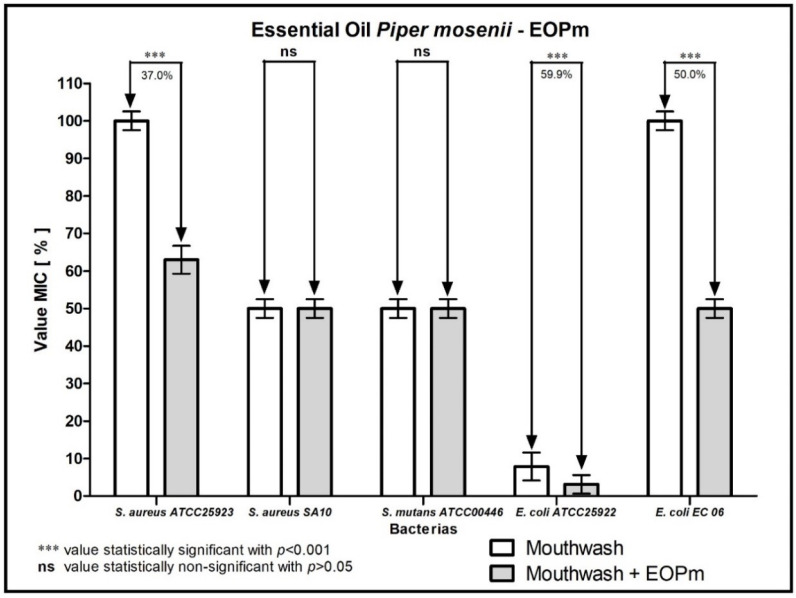
Evaluation of the modulating activity of EOPm combined with mouthwash against bacterial strains.

When analyzing the combination of EOPm with mouthwash (Figure 2), a 59.9% reduction in MIC was observed against the strain of *E. coli* ATCC25922. Against strains of *E. coli* EC06 and *S. aureus* ATCC25923, there was a decrease in 50% and 37% in MIC, respectively, indicating that there was a synergistic effect in this mentioned combination.

**Figure 3:**
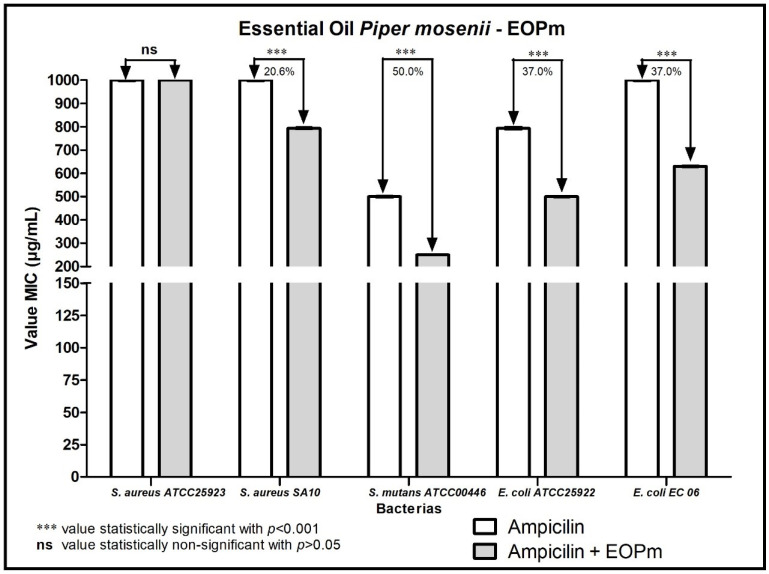
Evaluation of the modulatory activity of EOPm combined with Ampicillin against bacterial strains.

In the association of EOPm with the antibiotic ampicillin (Figure 3), it is possible to observe that there was a synergistic effect against the strains of *S. mutans* ATCC00446 and *S. aureus* SA10, considering that the MIC reduced 50% and 20.6%, respectively. Furthermore, this effect was also verified against *E. coli* EC06 and *E. coli* ATCC25922 strains, with a 37% decrease in ampicillin MIC for both.

**Figure 4:**
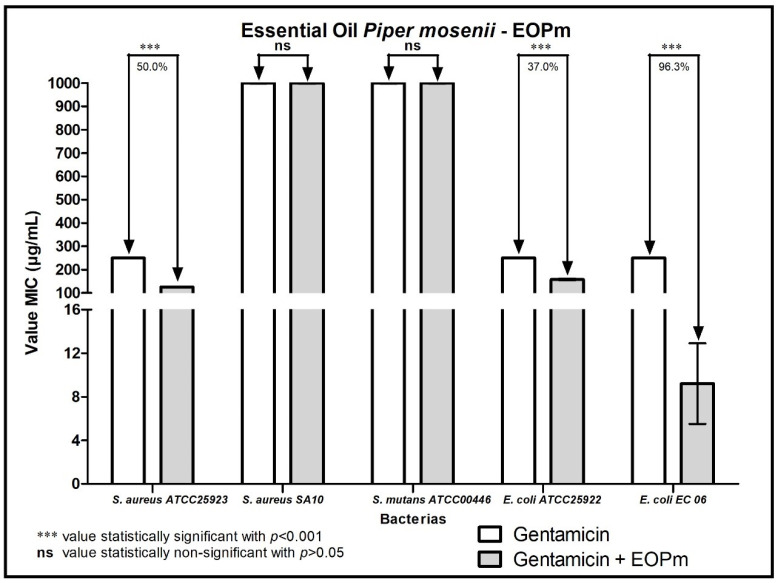
Evaluation of the modulatory activity of EOPa combined with Gentamicin against bacterial strains.

When the combination of EOPm with gentamicin was tested, there was a significant reduction in MIC against the *E. coli* EC06 strain, with a percentage of 96.3%, while for the strains of *S. aureus* ATCC25923 and *E. coli* ATCC25922 the decrease in MIC was of 50% and 37% respectively, also indicating that there was a synergistic effect (Figure 4).

**Figure 5:**
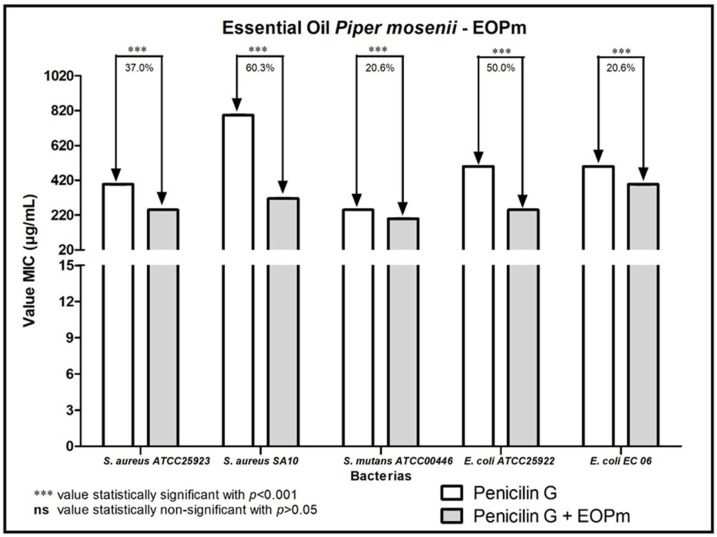
Evaluation of the modulatory activity of EOPm combined with Penicilin G against bacterial strains.

Figure 5 represents the result of the combination of the EOPm together with Penicillin G showed a reduction in MIC against all strains tested. Against the strain of *S. aureus* SA10, the reduction was 60.3%, while in the test of the association of products against the strain of *E. coli* ATCC25922 the reduction of the MIC was 50%. The *S. aureus* ATCC25923 strain, in turn, was inhibited with the MIC of the antibiotic reduced by 37%, while against *S. mutans* ATCC00446 and *E. coli* EC06 the combination showed lower percentages of MIC reduction, with 20.6%.

## 3. Discussion

As in antiquity, medicinal plants continue to play an important role as sources of therapeutic agents, originating herbal medicines or isolated active compounds that can be used as medicines or as prototypes for the production of new drugs. Substances of plant origin that have relevant pharmacological potential are, in general, secondary metabolites, belonging mainly to the classes of terpenes, coumarins, flavonoids and phenolic acids. Recent studies show that among the drugs approved between 1981 and 2014 for clinical use, 26% were natural products or derivatives and 13% had a pharmacophoric group of natural [18].

The terpenoid α-pinene, classified as a bicyclic monoterpene, is the main component of turpentine oil, obtained by distilling resins from pine trees (*Pinus* sp.) and other conifers, in addition to being found in the essential oil of several plants [19]. Some research has proven the biological properties of α-pinene isolated or contained in essential oils, in which they have antimicrobial, anti-inflammatory, antiallergic and antioxidant activity [20]. Studies performed with this compound provided an antibacterial effect against various strains, including *Escherichia coli* and *Staphylococcus aureus*, in addition to proving efficacy against pathogenic fungi and yeasts, such as *C. albicans* [21].

Myristicin, the second major chemical compound in EOPm, is commonly found in cinnamon, fennel, basil, cloves and especially in the seed of nutmeg (*Myristica fragrans*). It is used as a fragrance in the cosmetics industry and as a flavoring agent in foods. In addition, in traditional medicine, myristicin has been used in the treatment of cholera, stomach cramps, nausea, diarrhea, and anxiety. Recently, myristicin has been reported to exert antibacterial activity against Gram-positive and Gram-negative organisms, as well as having anti-inflammatory properties. However, its use in exaggerated amounts can induce toxic effects on the liver and central nervous system [22,23].

Although EOPm did not present antibacterial activity in this research, other studies have shown that natural products can be modulators of bacterial resistance when combined with antibiotics, enhancing their effect, and thus characterizing an alternative treatment against pathogenic microorganisms and a great ally to combat resistance microbial [24,25].

It is worth noting that in relation to the bacterium *Escherichia coli*, the emergence of resistance has recently been described, including to gentamicin, which is worrying because this species is the most common in infections caused by Gram-negative bacteria in humans [26]. However, when the EOPm modulating action with gentamicin was tested in this research, there was a significant synergistic effect for the two *Escherichia coli* strains.

The essential oil of *Piper mosenii* tested showed modifying action of antibiotics, chlorhexidine, and mouthwash, where EOPm presented more relevant results when associated, reducing the MIC of antibacterial substances. In this way, the modulation characteristics may be related to the phytochemical constituents contained in the essential oil through peculiarities of the bacterial cell wall [27].

## 4. Materials and Methods

### 4.1. Bacterial Strains

The pattern of bacterial strains used in the tests were *Staphylococcus aureus* (ATCC25923 and resistant SA10), *Escherichia coli* (ATCC25922 and resistant EC06) and *Streptococcus mutans* ATCC00446, which were provided by the Laboratory of Microbiology and Molecular Biology—LMBM, from the University Regional of Cariri—URCA, under the coordination of Prof. Dr. Henrique Douglas Melo Coutinho.

### 4.2. Preparation and Standardization of Bacterial Inoculo

Bacteria cultures were maintained at 4 °C in Heart Infusion Agar—HIA. Before testing, the strains were transferred to the HIA medium and incubated at 35 °C for 24 h. The active bacterial strains were inoculated in Brain Heart Infusion—BHI at the concentration recommended by the manufacturer and incubated under the same conditions mentioned above. Suspensions with bacterial growth were diluted in BHI at a concentration of 10% until obtaining 10^5^ cells/mL [28].

### 4.3. Antibiotics and Mouthwash Solutions

All substances used were dissolved in sterile water before use, which are: Chlorhexidine Digluconate, Gentamicin, Ampicillin and Penicillin G, were purchased from Sigma Chemical Corporation, St. Louis, MO, USA. The commercial mouthwash composed of water, glycerin, propylene glycol, sorbitol, tetrapotassium pyrophosphate, polysorbate 20, tetrasodium pyrophosphate, zinc citrate, PVM/MA copolymer, benzyl alcohol, sodium fluoride [225 ppm fluorine/0.05%], sodium saccharin, acid blue 3 [CI 42051]), was purchased at a drugstore.

### 4.4. Preparation of the Natural Product Obtained from Piper mosenii

#### 4.4.1. Obtaining the Essential Oil

The leaves of *Piper mosenii* C. DC. (P. mo.), were collected in the spring of 2019 in the Bom Jesus Biological Reserve (S 25°13.644′/W 48°34.985′), municipality of Guaraqueçaba, PR, Brazil. The specimens were deposited in the Herbarium of the Municipal Botanical Museum—MBM, in Curitiba, PR, Brazil, under number 396,412 (P.ar.). The plant material was collected through authorization from the Authorization and Information System on Biodiversity—SISBIO no. 49770-2. In addition, information about the species was registered in the National System for the Management of Genetic Heritage and Associated Traditional Knowledge (SISGEN) under the number A216E5A.

The leaves were dried, in the shade, at room temperature and submitted to hydrodistillation in a modified Clevenger-type apparatus [29] at the Laboratory of Chemistry and Biology of the Federal University of Paraná, Setor Costeiro. The oils were separated from the hydrolates with double-distilled dichloromethane, dried with anhydrous magnesium sulfate, filtered, concentrated on a rotary evaporator, transferred to a 5 mL flask, and stored in a refrigerator. The percentage of yield extraction was determined by the ratio between the mass of oil and the mass of plant material used (*w*/*w*), where 496 g of plant material produced 1.18 g of essential oil, with an approximate yield of 0.24%.

#### 4.4.2. Chemical Analysis of Essential Oil

GC-FID and GC-MS analysis of the essential oil sample of *Piper mosenii* (EOPm) were performed using a Shimadzu 14B GC equipped with a capillary column (DB5 Supelco, 30 µm × 0.25 µm id × 0.25 µm film thickness) and a Perkin-Elmer Clarus 680 equipped with a capillary column (DB5 Perkin Elmer, 30 µm × 0.25 µm id × 0.25 µm film thickness) attached to a Perkin-Elmer Clarus 600T, respectively.

In the GC-FID, the following analytical conditions were used: the injector and the detector operated at 250 °C and 280 °C, respectively. Helium carrier gas, 1 mL min^−1^ flow rate, 0.4 µL sample injection in split mode (1:20). The oven temperature was programmed from 60 °C (0 min) to 240 °C in a gradient of 3 °C min^−1^, being held at this temperature for 2 min, giving a total length analysis of 62 min. In the GC-MS, the following analytical conditions were used: sample injection (1.0 µL), helium carrier gas, flow rate 1 mL min^−1^, split mode (1:20), inlet temperature 220 °C, ion source at 250 °C and line transfer at 240 °C. The mass selective detector was set to 70 eV and a mass range of 40–400 amu. The oven temperature was programmed from 60 °C (0 min) to 246 °C in a gradient of 3 °C min^−1^, giving a full-length analysis of 62 min.

The identification of components was performed by searching a computer library based on MS spectra pairing, comparison with literature data [30] and experimental arithmetic indices (AI) [31], which were calculated using a homologous series of linear alkanes analyzed under the same conditions as the GC flame ionization detector (FID) described above. Component quantification was based on their GC peak areas without correction for response factors.

### 4.5. Antibacterial Assays

#### 4.5.1. Determination of Minimum Inhibitory Concentration (MIC) In Vitro by Direct Contact

Assays to determine the MIC of essential oil of *Piper mosenii* (EOPm) [1024 µg/mL], antibiotics (ampicillin, gentamicin, penicillin G) [1000 µg/mL], chlorhexidine (CLX) [0.06%], and commercial mouthwash [100%] were performed using the Broth Microdilition Method, with concentrations ranging from [C_initial_/2] to [C_initial_/11]. Bacterial suspensions were diluted 1:10 in BHI Broth to obtain a final concentration of 10^5^ cells/mL [28]. Test product samples were prepared in double concentration, where the initial concentrations (C_initial_) were: EOPm [1024 µg/mL], antibiotics [1000 µg/mL], chlorhexidine (CLX) [0.06%], and mouthwash commercial buccal (EN) [100%] in relation to initial concentration and volumes of 100 µL will be serially diluted 1:1 in 10% BHI broth. In each well with 100 µL of the culture medium a sample of bacterial suspension diluted 1:10. Negative controls with the culture medium, positive controls (medium + inoculum) and inhibition controls using the tested products were included in the assays. The filled plates will be incubated at 35 °C for 24 h [28]. To demonstrate the MIC of the samples, an indicator solution of sodium resazurin (Sigma) in sterile distilled water at a concentration of 0.01% (*w*/*v*) was used. After incubation, 20 µL of the indicator solution was added to each well and the plates were incubated for 1 h at room temperature. The change from blue to pink color due to the reduction of resazurin indicated bacterial growth [32], helping to visualize the MIC, defined as the lowest concentration capable of inhibiting microbial growth, evidenced by the unchanged blue color.

#### 4.5.2. Modulating Activity of Antibiotic Action In Vitro by Direct Contact

To evaluate *P.* *mosenii* essential oil (EOPm) as a modulator of the antibacterial action of antibiotics (ampicillin, gentamicin, penicillin G) [1000 µg/mL], chlorhexidine (CLX) [0.06%] and commercial mouthwash (EN) 100%, MIC was evaluated in the presence and absence of EOPm in sterile 96-well microplates.

EOPm was mixed in 10% BHI broth at subinhibitory concentrations (MIC/8). Antibiotic solutions were prepared with sterile distilled water in double concentration (1000 µg/mL) in relation to the defined initial concentration and volumes of 100 µL will be serially diluted 1:1 in 10% BHI broth. Each well with 100 µL of the culture medium contains the diluted bacterial suspension (1:10). The same controls used in the MIC assessment for the test products were used. The filled plates will be incubated at 35 °C for 24 h and the reading was evidenced by the use of sodium resazurin as mentioned above.

### 4.6. Statistical Analysis of Microbiological Assays

The MIC results obtained in triplicate in the bacterial resistance modulation tests will be tabulated in a spreadsheet using Microsoft Excel 2010 software, and applying the geometric mean formula and deviation calculation, obtaining parametric data and possible submission to statistical analysis and significance test.

For statistical analysis, the data expressed as the geometric mean ± standard error of the mean (SEM) were submitted to analysis of variance (ANOVA), followed by the Bonferroni significance test, considering a significant difference when *p* < 0.001, using the Prisma 5 for Windows Version 5.02 (GraphPad Software, San Diego, CA, USA) Software.

## 5. Conclusions

In view of the results presented, the chemical composition of the essential oil obtained from the leaves of *Piper mosenii* contains substances belonging to chemical classes with proven biological activity, but the antibacterial activity of EOPm demonstrated clinically irrelevant potential. However, when EOPm was associated with chlorhexidine, mouthwash, and antibiotics (ampicillin, gentamicin, and penicillin G) to assess its influence on bacterial resistance, the oil showed significant synergistic activity, reducing the MIC of the tested products from 20.6% to 96%. Therefore, it is suggested the expansion of tests with greater variation of combinations of EOPm concentration and the products used in this research, as well as evaluation of toxicity and in vivo tests, with the objective of developing a possible formulation of a low-cost mouthwash accessible to the most vulnerable population.

## Figures and Tables

**Table 1:** GC-MS analysis of the chemical composition of essential oil *Piper mosenii* (EOPm).

No.	Constituents	RT (min)GC-MS (EOPm)	IA_exp_	IA_lit_	EOPm [%]
1	α-pinene	5.860	929	932	22.8
2	β-pinene	7.100	976	974	3.8
3	Myrcene	7.390	985	988	0.7
4	Limonene	8.740	1027	1024	1.1
5	1,8-cineole	8.871	1031	1026	<0.1
6	Linalool	11.360	1096	1095	0.5
7	α-copaene	22.870	1374	1374	1.2
8	β-elemene	23.450	1387	1389	0.6
9	β-caryophyllene	24.656	1419	1417	6.1
10	Aromadendrene	25.410	1437	1439	2.8
11	α-caryophyllene	26.103	1454	1452	3.1
12	allo-aromadendrene	26.270	1459	1458	1.6
13	γ-muurolene	27.140	1480	1478	1.0
14	β-selinene	27.440	1487	1489	2.1
15	Bicyclogermacrene	27.710	1495	1500	8.3
16	Myristicin	28.720	1517	1517	16.5
17	(*E*)-nerolidol	30.340	1557	1561	1.2
18	Spathulenol	30.840	1577	1577	3.4
19	Caryophyllene oxide	31.040	1582	1582	3.5
20	Globulol	31.180	1585	1590	2.4
21	Ledol	31.890	1604	1602	1.0
22	1,2-humulene epoxide	32.080	1609	1608	2.1
23	4-heptyl-acetophenone *	34.370	1672	-	1.4
	**Total**				**87.20**

IA_exp_: Experimental arithmetic retention index; IA_lit_: Arithmetic retention index from the literature [17]. * Tentatively identified. Structural elucidation based on the mass spectrum. The data presented in the table above were produced by analyzing the sample using gas chromatography techniques coupled to flame ionization detectors (GC-DIC) and mass (GC-MS). The arithmetic retention index was determined using linear patterns of saturated hydrocarbons (C8–C19 carbon number).

## Data Availability

Not applicable.

## Sample Availability

Samples of compounds, results and corrections have been shared and are available with all authors and confirmed for publication.
